# Post-Coronavirus Disease 2019 Syndrome in Japan: An Observational Study Using a Medical Database

**DOI:** 10.31662/jmaj.2023-0048

**Published:** 2023-07-18

**Authors:** Yasuha Kinugasa, Mara Anais Llamas-Covarrubias, Katsuhiko Ozaki, Yoshiaki Fujimura, Takeki Ohashi, Kou Fukuda, Shinichi Higashiue, Yusuke Nakamura, Yumiko Imai

**Affiliations:** 1 National Institutes of Biomedical Innovation, Health and Nutrition (NIBIOHN), Ibaraki, Japan; 2 Tokushukai Information System, Osaka, Japan; 3 Tokushukai Group, Osaka, Japan

**Keywords:** COVID-19, post-COVID-19 syndrome, electronic health records, vaccination, virus mutation, older adult population, nursing care, quality of life

## Abstract

**Introduction:**

In Japan, the clinical information on post-COVID-19 syndrome, including nursing care requirements, is limited. The present study investigated the incidence of acute and post-COVID-19 symptoms, including nursing care requirements, when different SARS-CoV2 strains were prevalent and vaccination statuses changed to mass vaccination programs in Japan.

**Methods:**

Electronic health records of 122,045 patients diagnosed with COVID-19 between January 1, 2020, and June 30, 2022, were obtained from the Tokushukai Group Medical Database. Patient data was divided into three observation periods. Using the International Statistical Classification of Diseases and Related Health Problems 10 codes, typical symptoms of acute (within two weeks after diagnosis) and post-COVID-19 (2-12 weeks after diagnosis) were extracted. Moreover, the nursing care requirements of patients who visited the hospital before and after the COVID-19 diagnosis were examined.

**Results:**

Original and alpha strains were prevalent in Period 1, wherein most of the population was unvaccinated. The delta strain was prevalent in Period 2, wherein approximately 70% of the population was vaccinated. The omicron strain was prevalent in Period 3, wherein approximately 70% of the population completed the two vaccination doses. Headache, malaise/fatigue, depression, and disuse syndrome were detected in acute and post-COVID-19. The incidence of depression and disuse syndrome in post-COVID-19 increased with age, with the highest incidence in the 60-85-year group. Moreover, increased high-level nursing care requirements were observed after COVID-19 in the 60-85-year-age group.

**Conclusions:**

A lower incidence of acute and post-COVID-19 symptoms in Japan is linked to increased population vaccination coverage. However, differences in viral strains may be involved. Moreover, a reduction in long-term quality of life exists in older adult patients after COVID-19. These data provide fundamental information for preventing and treating post-COVID-19 syndrome in Japan.

## Introduction

Although most patients with coronavirus disease 2019 (COVID-19) recover within a few weeks after acute pulmonary infection; some patients exhibit various symptoms in multiple organs lasting more than two months. These symptoms are collectively termed post-COVID-19 syndrome or long COVID ^(1)^. However, these terms lack a unified definition. The World Health Organization (WHO)’s clinical case definition is “post-COVID-19 condition occurs in individuals with a history of probable or confirmed severe acute respiratory syndrome-coronavirus 2 (SARS-CoV-2) infection, usually three months from the onset, with symptoms that last for at least two months and cannot be explained by an alternative diagnosis ^(2)^”. Several worldwide long COVID cohort studies and systematic reviews, although heterogeneous, show that more than 200 COVID-19 symptoms persist beyond the acute phase of infection and affect health-related functions and life quality ^(3), (4), (5), (6), (7), (8), (9)^. A comprehensive review of the effects of long COVID on various organ systems has recommended rehabilitation plans for COVID-19 survivors ^(10)^. However, information regarding the pathophysiology of long COVID is limited and controversial. Furthermore, optimal prevention and treatment strategies for COVID-19 are yet to be developed.

In Japan, more than 33.3 million people have been infected with SARS-CoV2, and more than 72,000 have died of COVID-19 as of March 4, 2023 ^(11)^; however, clinical information on post-COVID-19 syndrome is limited. Since the COVID-19 outbreak, Japan has experienced COVID-19 epidemics caused by different mutant strains of SARS-CoV2, such as alpha, delta, and omicron ^(12)^. Mass vaccination programs conducted during epidemic periods have substantially changed the vaccination status of the population ^(13)^. However, the effect of viral strains and vaccination status on developing long COVID and nursing care requirements remains unclear, and some reports exist with inconsistent results ^(11), (14), (15)^.

The Tokushukai Group operates 73 hospitals and 340 primary care clinics throughout Japan. Currently, medical information based on electronic health records (EHRs) of more than 13 million patients is compiled in the Tokushukai Group Medical Database operated by the Tokushukai Information System (TIS). In the present study, using the structured EHRs in TIS on approximately 120,000 patients with COVID-19, we investigated the incidence of acute and post-COVID-19 symptoms (i.e., prevalence relative to the timing after COVID-19) and nursing care requirements when different virus strains were prevalent and vaccination statuses were also changed in Japan.

## Materials and Methods

### Participants and collection of EHRs

The study was approved by the Ethics Committee of the research institutions involved (293m), and an opt-out system was used instead of obtaining informed consent from the patients. We collected the TIS’s structured EHRs of inpatients and outpatients (total n = 122,045) diagnosed with COVID-19 between January 1, 2020, and June 30, 2022. COVID-19 was diagnosed using polymerase chain reaction for SARS-CoV2, and the International Statistical Classification of Diseases and Related Health Problems 10 (ICD-10) = U071/U072 was recorded in the EHRs once the diagnosis was confirmed. We included the incomplete EHRs (e.g., mortality, loss-to-follow-up) ^(16)^. We divided the patient data into three observation periods: Period 1 (January 1, 2020, to June 30, 2021; n = 17,128), Period 2 (July 1, 2021, to December 31, 2021; n = 17,873), and Period 3 (January 1, 2022, to June 30, 2022; n = 87,044).

### Incidence of acute and post-COVID-19 symptoms

We extracted data from patients with symptoms within three months of COVID-19 diagnosis but did not show symptoms before COVID-19, separately for acute COVID-19 (within two weeks) and post-COVID-19 (2-12 weeks), wherein replication-competent SARS-CoV2 latter period had not yet been isolated ^(17)^. We used the following ICD-10 codes: R51 and G44 for headache; R53 for malaise/fatigue; M625 for disuse syndrome; F3, F4, and F5 for depression; R432 for taste disorder; R430 and R431 for olfactory disorder; and L63-L66 for alopecia. The data were classified into three age categories: 0-19, 20-59, and 60-85 years.

### Nursing care requirements

We examined the effect of COVID-19 on the nursing care of patients with COVID-19 who visited Tokushukai Group Hospitals 380 days before and after COVID-19 diagnosis (n = 534). The nursing care levels were certified by the government and classified into seven levels ^(18)^. Information on nursing care levels was extracted from the diagnosis procedure combination data of the TIS medical database for patients with COVID-19 aged 50-85 years. We further classified the seven levels into three groups: “support 1 and 2” (mild level which only required support), “care 1 to 3” (moderate level which required nursing care for some daily activities), and “care 4 and 5” (severe level which required nursing care for almost all activities).

### Statistical analysis

All data were analyzed using the chi-square test or Fisher’s exact test. The Fisher’s exact test was applied to groups with five or fewer patients. To analyze the incidence of acute and post-COVID-19 symptoms, Bonferroni correction was performed following the chi-square test or Fisher’s exact test to correct errors in multiple testing. Excel (Microsoft, WA, USA) and GraphPad Prism 9 (GraphPad Software, Boston, MA) were used for the analyses. A value of *α* = 0.05 indicated statistical significance, and *α* = 0.1 was considered a significance level with tendency.

## Results

### Patient population profiles

**Figure 1.**
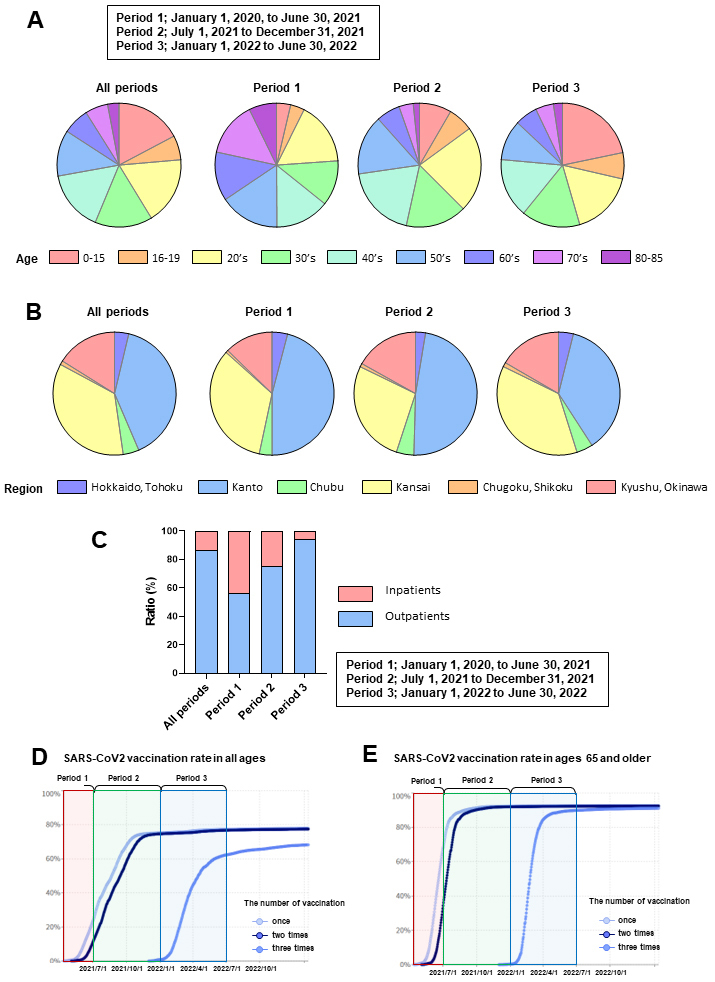
Target patients and periods of this study A. Percentage of patients according to age, divided into Period 1 (January 1, 2020, to June 30, 2021), 2 (July 1, 2021, to December 31, 2021), and 3 (January 1, 2022, to June 30, 2022). B. Percentage of patients according to region, divided into Periods 1, 2, and 3. C. Percentage of outpatients and inpatients divided into Periods 1, 2, and 3. D. Domestic SARS-CoV2 vaccination rates in individuals of all ages, and E. those aged 65 years and older from January 2021 to February 2023 according to the number of vaccinations (data obtained from a government digital agency ^(13)^ have been modified).

A total of 122,045 patients diagnosed with COVID-19 between January 1, 2020, and June 30, 2022, were enrolled in this study. The numbers (Table 1) and percentages (Figure 1A) of patients by age, divided into periods 1, 2, and 3, are shown. The numbers (Table 2) and percentages (Figure 1B) of patients by local regions are presented. The number (Table 3) and percentage (Figure 1C) of outpatients and inpatients are shown. According to a report from the National Institute of Infectious Diseases ^(19)^, the original and alpha strains were prevalent during observation in Period 1, followed by the delta and omicron strains in Periods 2 and 3, respectively. Notably, the domestic SARS-CoV2 vaccination rates for individuals of all ages (Figure 1D) and those aged 65 years and older (Figure 1E) from January 2021 to February 2023 were reported by a digital agency ^(13)^. Considering these factors, the observation Period 1 epidemic was mainly caused by the original or alpha strain, wherein most of the population was unvaccinated. The epidemic during observation Period 2 was caused primarily by the delta strain, and approximately 70% of the population (approximately 90% of those aged 65 years and older) was vaccinated. The epidemic during observation Period 3 was caused primarily by the omicron strain, in which approximately 70% of the population (approximately 90% of those aged 65 years and older) had completed two vaccination doses.

**Table 1.** Number of Eligible Patients with COVID-19 by Age, Divided into Observation Periods to Examine Acute and Post-COVID-19 Incidence.

	Age	0-15	16-19	20’s	30’s	40’s	50’s	60’s	70’s	80-85	Total
Observation periods	Period 1; January 1, 2020-June 30, 2021	635	627	2,829	2,023	2,433	2,695	2,185	2,453	1,248	17,128 (14.0%)
	Period 2; July 1, 2021-December 31, 2021	1,504	1,176	4,008	2,868	3,440	2,791	1,118	669	299	17,873 (14.6%)
	Period 3; January 1, 2022-June 30, 2022	18,930	5,960	14,702	13,411	13,517	9,164	5,133	4,141	2,086	87,044 (71.3%)
	Total	21,069	7,763	21,539	18,302	19,390	14,650	8,436	7,263	3,633	122,045 (100.0%)

**Table 2.** Number of Eligible Patients with COVID-19 by Region, Divided into Observation Periods to Examine Acute and Post-COVID-19 Incidence.

	Region	Hokkaido, Tohoku	Kanto	Chubu	Kansai	Chugoku, Shikoku	Kyushu, Okinawa	Total
Observation periods	Period 1; January 1, 2020-June 30, 2021	691	7,870	572	5,713	114	2,168	17,128 (14.0%)
	Period 2; July 1, 2021-December 31, 2021	467	8,548	823	4,851	165	3,019	17,873 (14.6%)
	Period 3; January 1, 2022-June 30, 2022	3,388	32,186	3,721	32,290	987	14,472	87,044 (71.3%)
	Total	4,546	48,604	5,116	42,854	1,266	19,659	122,045
		(3.7%)	(39.8%)	(4.2%)	(35.1%)	(1.0%)	(16.1%)	(100.0%)

*The regional information was obtained from the hospital address.

**Table 3.** The Number of Eligible Outpatients/Inpatients with COVID-19, Divided into Observation Periods to Examine Acute and Post-COVID-19 Incidence.

	Out/Inpatients	Outpatients	Inpatients	Total
Observation periods	Period 1; January 1, 2020-June 30, 2021	9,609	7,519	17,128 (14.0%)
	Period 2; July 1, 2021-December 31, 2021	13,401	4,472	17,873 (14.6%)
	Period 3; January 1, 2022-June 30, 2022	82,220	4,824	87,044 (71.3%)
	Total	105,230	16,815	122,045
		(86.2%)	(13.8%)	(100.0%)

### Occurrence of acute and post-COVID-19 symptoms

**Figure 2.**
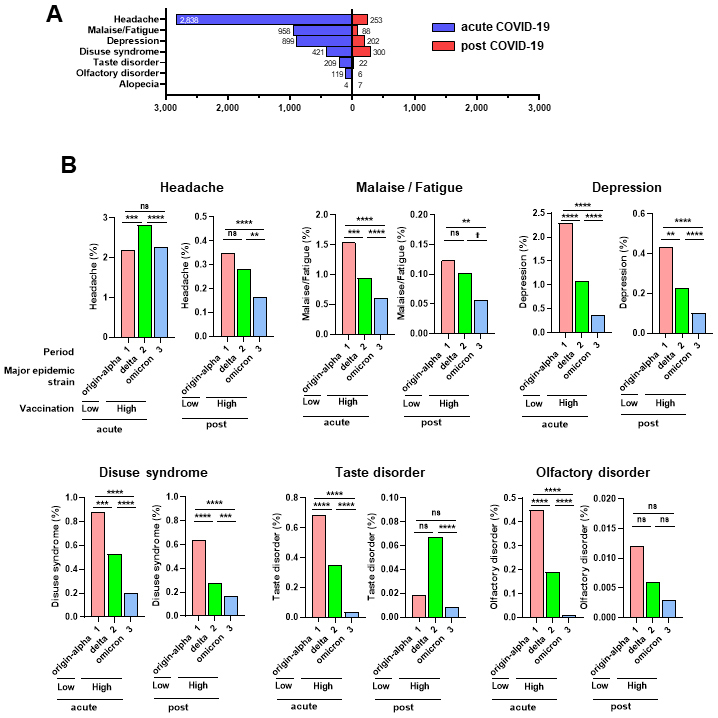
Incidence of acute and post-COVID-19 symptoms in epidemic periods A. Number of headaches, malaise/fatigue, depression, disuse syndrome, taste disorder, olfactory disorder, and alopecia cases in acute (within two weeks of diagnosis) and post-COVID-19 (2-12 weeks after diagnosis). The numbers are displayed at the tip of the bars. B. Incidence of acute and post-COVID-19 symptoms in three observation periods, along with major epidemic strain and vaccination status. The *p*-value was calculated using the chi-squared or Fisher’s exact tests and then compared with a corrected significance level using Bonferroni’s method (*α*′). Asterisks and a dagger indicate that the *p*-value between the two groups was less than *α*′ using different original significance level (*α*); **** *α* = 0.0001, *** *α* = 0.001, ** *α* = 0.01, * *α* = 0.05, and † *α* = 0.1. ns represents no significance.

The symptoms were classified as acute (within two weeks of COVID-19 diagnosis) and post-COVID-19 (2-12 weeks after COVID-19 diagnosis). Headache, malaise/fatigue, depression, and disuse syndrome were detected post-COVID-19 but were also common in the acute phase of COVID-19, suggesting that these symptoms can develop in the acute phase and persist thereafter (Figure 2A). Depression and disuse syndrome frequently occurred post-COVID-19 (Figure 2A). In contrast, taste and olfactory disturbances occurred mainly in patients with acute COVID-19; however, these were also persistently detected post-COVID-19, albeit in a small number of patients (Figure 2A). The number of patients with alopecia was small (n = 4 for acute COVID-19 and n = 7 for post-COVID-19).

Next, we examined the occurrence rates of symptoms during all and three observation periods. Post-COVID-19, the frequency of headaches, malaise/fatigue, depression, and disuse syndrome were significantly lower in Period 3 than in Period 1. Additionally, the frequency of headaches, depression, and disuse syndrome were significantly lower in Period 3 than in Period 2 (Figure 2B). During the acute COVID-19 phase, except for headache, the frequency of other symptoms was significantly lower in Period 2 than in Period 1, and their frequencies in Period 3 were lower than in Period 2 (Figure 2B). Overall, our data suggest that increased vaccination coverage of the population may reduce the incidence of COVID symptoms, particularly headache, malaise/fatigue, depression, and disuse syndrome, in the acute phase and post-COVID. However, differences in virulence due to mutations during the observation period cannot be ruled out.

**Figure 3.**
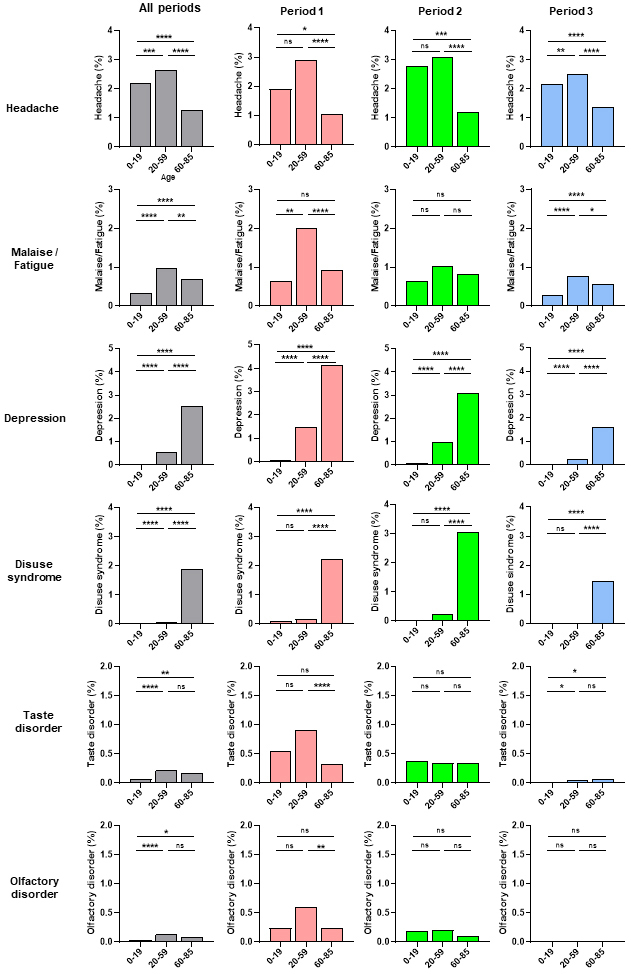
Incidence of acute COVID-19 symptoms according to age The incidence of acute COVID-19 symptoms in patients with COVID-19 aged 0-19, 20-59, and 60-85 years in all three observation periods. The *p*-value between the two groups was calculated using the chi-squared or Fisher’s exact tests and then compared with a corrected significance level using Bonferroni’s method (*α*′). Asterisks indicate that the *p*-value was less than *α*′ using different original significance level (*α*); **** *α* = 0.0001, *** *α* = 0.001, ** *α* = 0.01, and * *α* = 0.05. ns indicates no significant difference.

**Figure 4.**
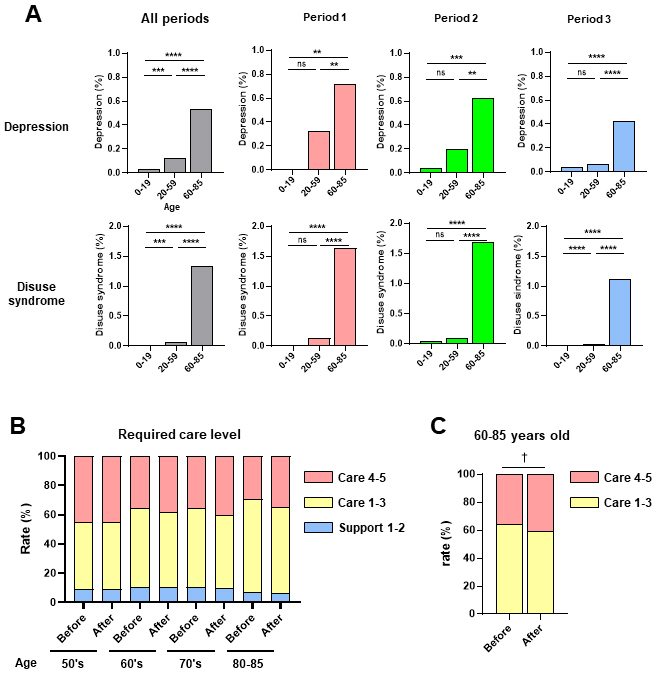
Incidence of post-COVID-19 symptoms and changes in required care before and after COVID-19. A. Incidence of post-COVID-19 symptoms of depression and disuse syndrome in patients with COVID-19 aged 0-19, 20-59, and 60-85 years in three observation periods. The *p*-value between the two groups was calculated using the chi-squared or Fisher’s exact tests and then compared with a corrected significance level using Bonferroni’s method (*α*′). Asterisks indicate that the *p*-value was less than *α*′ using different original significance level (*α*); **** *α* = 0.0001, *** *α* = 0.001, and ** *α* = 0.01. ns represents no significance. B. Changes in nursing care requirements according to age before and after COVID-19 for support levels 1-2, care levels 1-3, and care levels 4-5. C. Changes in nursing care requirements of ages 60-85 years before and after COVID-19 for care levels 1-3, and care levels 4-5. The *p*-value between the two groups was calculated using the chi-squared test and then compared with a corrected significance level using Bonferroni’s method (*α*′). A dagger indicates that the *p*-value was less than *α*′ using a different original significance level (*α*); † *α* = 0.1. represents tendency.

Next, we examined the frequency of acute COVID-19 symptoms according to age during the three observation periods (Figure 3). Headache and malaise/fatigue occurrence rates were significantly higher in the middle-age (20-59 years old) group than in the young-age (0-19 years old) or old-age (60-85 years old) groups, with a similar trend observed in the three periods (Figure 3). In contrast, depression and disuse syndrome occurrence rates were significantly higher in the old-age group than in the middle- or young-age groups, with the same trend in all three periods (Figure 3). Although the number of cases was small, taste and olfactory disorders occurrence rates did not significantly differ according to age or period (Figure 3). Depression and disuse syndrome occurred more frequently than other symptoms in post-COVID-19 (Figure 2A). Next, we examined the frequency of depression and disuse syndromes post-COVID-19 according to age during the three periods (Figure 4A). Depression and disuse syndrome occurrence rates significantly increased with age and were highest in the old-age group, with the same trend in all three periods (Figure 4A). These findings suggest that the occurrence of headache and malaise/fatigue, particularly in acute COVID-19, was significantly higher in the middle-aged group than in the young- or old-age groups, whereas the occurrence of depression and disuse syndrome, particularly post-COVID-19, increased with age and was highest in the old-age group.

### Changes in the level of nursing care requirements

Using the TIS’s structured EHRs, we extracted data of patients who visited Tokushukai Group Hospitals 380 days before and after COVID-19 diagnosis (n = 534). Patient profiles according to observation period and age (>50 years) are shown in Table 4. Nursing care levels were certified by the government and classified into seven levels ^(18)^; support required 1-2 and care required 1-5. We further classified the seven levels into three groups: Support 1 and 2 (mild level which only required support for daily activities and social worker visits once or twice a week), care 1-3 (moderate level which required nursing care for some daily activities and social worker visits three days a week), and care 4-5 severe levels (which required nursing care for almost all activities and social worker visits six days a week). The changes in nursing care requirements according to age before and after COVID-19 for support levels 1-2, care levels 1-3, and care levels 4-5 are shown in Figure 4B. There was a trend toward an increase in the percentage of severe care requirements (care levels 4-5) after COVID in individuals aged 60-85 years (Figure 4C). These findings suggest that COVID-19 exacerbates nursing care requirements, especially in older adults.

**Table 4.** Number of Eligible Patients with COVID-19 by Age (>50 Years), Divided into Observation Periods for Examination of Nursing Care Requirements.

	Ages over than 50’s	50’s	60’s	70’s	80-85	Total
Observation periods	Period 1; January 1, 2020-June 30, 2021	1	10	67	74	154 (28.8%)
	Period 2; July 1, 2021-December 31, 2021	0	5	21	22	48 (9.0%)
	Period 3; January 1, 2022-June 30, 2022	10	24	131	166	332 (62.2%)
	Total	11	39	219	262	534 (100%)

## Discussion

Using structured EHRs on approximately 120,000 patients with COVID-19, we demonstrated that some of the major symptoms of COVID-19 (e.g., headache, malaise/fatigue, depression, and disuse syndrome) developed during the acute phase and persisted thereafter as long COVID. Increased vaccination coverage through mass vaccination programs has decreased the incidence of these symptoms. As the incidence of these symptoms was lower in Period 3 than in Period 2, differences in the virulence of the viral strains due to mutations during the epidemic period cannot be ruled out. Notably, diagnostic methods for viral strains varied between periods, from test sensitivity to viral factors, such as S gene, dropout due to the 69-70del mutation in alpha and omicron, except delta. This may have led to the underdiagnosis of certain strains during a certain pandemic period. Several studies have reported that vaccination is involved in the onset of long COVID. A meta-analysis of data from public databases showed that vaccination tends to decrease the sequelae of COVID-19 ^(14), (20)^. Regarding differences in virus strains, a large-scale self-reported smartphone survey of approximately 50,000 (vaccinated) long COVID cases from the United Kingdom reported a higher risk of developing long COVID during the delta strain epidemic than during the omicron strain epidemic ^(21)^.

In the present study, the incidence of depression and disuse syndrome increased with age and was highest in the old-age group. We also found a trend toward an increase in high-level nursing care requirements after COVID in the 60-85 years group, suggesting that the onset of depression and disuse syndrome in older individuals after COVID-19 may be linked to their long-term quality of life (QOL). In contrast, compared to those in Periods 2 and 3, depression and disuse syndrome in elderly patients were much more prevalent in Period 1, indicating that a stringent lockdown may limit their movement and potentially prohibit families from visiting a nursing home. Such causes may correlate with the incidence of depression and disuse syndrome. Prolonged self-restraint due to the pandemic, such as decreased physical activity and communication, may influence lifestyle, especially in older individuals, increasing their risk of decreasing QOL. To clarify the contribution of these factors to changes in QOL, especially in the elderly, further studies should include non-COVID-19 patients as controls during the same period, since these symptoms may also appear in non-COVID-19 patients due to COVID-19-associated changes in social life.

COVID-19 affects multiple organs and has a broad spectrum of manifestations. During the long-term course of COVID-19, complex interactions between the viral and host systems (e.g., the immune, cardiovascular, and nervous systems) can, directly and indirectly, affect multiple organs ^(1)^, in which host genetic and epigenetic factors may be involved. Although mechanistic studies are generally in the early stages of the disease, several hypotheses for the long COVID pathogenesis have been suggested, including the presence of persistent reservoirs of SARS-CoV-2 in tissues ^(22)^, immune dysregulation ^(22), (23)^, effects of SARS-CoV-2 on the microbiota ^(24)^, involvement of autoimmunity ^(25)^, microvascular blood clotting with endothelial dysfunction ^(26)^, and dysfunctional signaling in the nervous system ^(27)^. Potential risk factors include female sex, type 2 diabetes, Epstein-Barr virus reactivation, and the presence of specific autoantibodies ^(28)^.

This study has several limitations. In the present study, the patients were followed up for three months after COVID-19, but it is important to observe them for a longer period, from one to several years. In addition, this study focused on limited medical information. Several critical analyses, such as the vaccination status of the individual patient, other symptoms (e.g., gastrointestinal and heart problems), treatment (e.g., corticosteroids and antiviral drugs), inpatient/outpatient, and regions, are not shown in the present study, and future data-driven research using artificial intelligence and other methods at multimodal endpoints is needed. Also, incomplete cases, such as mortality or loss-to follow-up, were included, although the number of such cases are small. Moreover, we showed symptoms within three months of COVID-19 diagnosis, but not before COVID-19, separately for acute and post-COVID-19, so-called symptom prevalence relative to the timing after COVID-19. Thus, this study did not examine alternative diagnoses or chronic diseases after the COVID-19 diagnosis, as described in the WHO definition of long COVID clinical cases. We believe that further detailed investigations under the WHO’s long COVID clinical case definition are warranted.

To this end, accelerating the digital medical transformation is urgent to enable the sharing of health, medical, and nursing care information and the reviewing and utilization of personal health data by individuals. Despite several limitations, these data on the Japanese population can provide basic information that can lead to the long-term prevention and treatment of long COVID.

## Article Information

### Conflicts of Interest

None

### Sources of Funding

This work was partially supported by JSPS KAKENHI (grant numbers: 17H06179, 17K19693, and 15H05978).

### Acknowledgement

We thank the Tokushukai Group Hospitals for cooperating in this study and all laboratory members for their helpful discussions. We also thank Editage (www.editage.com) for English language editing.

### Author Contributions

YK, ML, YN, and YI conceived the study topic. KO, YF, TO, KH, and SH contributed to clinical information collection. YK, YI, KO, and YF contributed to the data analysis. YK, ML, YI, and YN drafted the manuscript. All authors critically reviewed and revised the manuscript draft and approved the final version for submission.

### Approval by Institutional Review Board (IRB)

293m (National Institutes of Biomedical Innovation, Health and Nutrition)
